# Association between Maternal Pelvis Height and Intrapartum Foetal Head Moulding in Ugandan Mothers with Spontaneous Vertex Deliveries

**DOI:** 10.1155/2016/3815295

**Published:** 2016-02-29

**Authors:** Ian G. Munabi, Samuel Abilemech Luboga, Livingstone Luboobi, Florence Mirembe

**Affiliations:** ^1^Department of Human Anatomy, School of Biomedical Sciences, Makerere University College of Health Sciences, New Mulago Hospital Complex, Kampala, Uganda; ^2^Department of Mathematics, Makerere University College of Natural Sciences, Makerere University, Kampala, Uganda; ^3^Department of Obstetrics and Gynaecology, School of Medicine, Makerere University College of Health Sciences, New Mulago Hospital Complex, Kampala, Uganda

## Abstract

*Introduction*. In Sub-Saharan Africa, excessive foetal head moulding is commonly associated with cephalopelvic disproportion and obstructed labour. This study set out to determine the associations of maternal pelvis height and maternal height with intrapartum foetal head moulding.* Methods*. This was a multisite secondary analysis of maternal birth records of mothers with singleton pregnancies ending in a spontaneous vertex delivery. A summary of the details of the pregnancy and delivery records were reviewed and analysed using multilevel logistic regression respect to foetal head moulding. The alpha level was set at *P* < 0.05.* Results*. 412 records were obtained, of which 108/385 (28%) observed foetal head moulding. There was a significant reduction in risk of foetal head moulding with increasing maternal height (Adj. IRR 0.97, *P* = 0.05), maternal pelvis height (Adj. IRR 0.88, *P* < 0.01), and raptured membranes (Adj. IRR 0.10, *P* < 0.01). There was a significant increased risk of foetal head moulding with increasing birth weight (Adj. IRR 1.90, *P* < 0.01) and duration of monitored active labour (Adj. IRR 1.21, *P* < 0.01) in the final model.* Conclusion*. This study showed that increasing maternal height and maternal pelvis height were associated with a significant reduction in intrapartum foetal head moulding.

## 1. Introduction

Foetal head moulding has been described as one of the responses of the foetus to cephalopelvic disproportion (CPD) associated with childbirth [1]. CPD is observed when there is a miss match between the diameters of the presenting part of the foetal head and the diameters of the maternal birth canal [2]. This miss match results in a failure of labour to progress despite the continued maternal effective and sometimes strong uterine contractions. Over time this continued maternal effort leads to foetal hypoxia as a result of progressive reduction in maternal arterial placental blood supply during the long uterine contractions and maternal exhaustion [1–3].

The human female pelvis is prone to CPD as explained by its evolutionary description and adaptation between the bipedal gait and the need to allow the passage of a large brained foetus [4]. Malnutrition further aggravates the occurrence of cephalopelvic disproportion by leading to loss in bone development in the growing mothers, resulting in small pelvic diameters [5]. Mothers who have been exposed to malnutrition as children may in turn as adults feed well to carry normal or big sized foetuses thus setting the scene for CPD. Excessive foetal head moulding as a result of CPD is one of the events in prolonged labour that may later become obstructed [1, 2].

**Figure 1:**
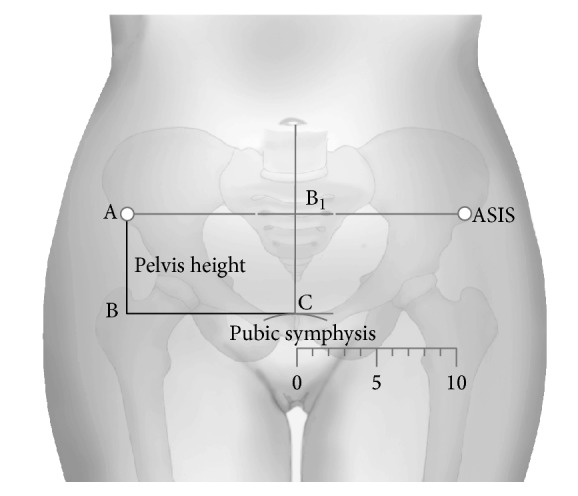
Measurement of pelvis height.

Obstructed labour as a result of CPD remains common in Sub-Saharan Africa [6]. In this region chronic malnutrition in combination with recurrent infections and early pregnancies have been identified as some of the predisposing factors for CPD [5, 7–9]. The observation that nutritional interventions take more than one generation to correct growth deficiencies [5] points to a need for further identification of easy to use appropriate evidence based tools for screening mothers with the potential to develop CPD in low resource settings [10]. Maternal height remains a good indicator of chronic malnutrition even though it is not one of the WHO recommended antenatal screening assessments [11–14]. In this paper we explore the use of maternal pelvis height as an additional anthropometric measurement to predict foetal head moulding in low resource settings. Pelvis height is currently used by automotive vehicle engineers to mark off the contribution pelvis bones to total height in crash test dummies [15]. Pelvis height is obtained using two easy to identify bony landmarks (symphysis pubis and anterior superior iliac spines) as shown in Figure 1 with mothers lying in the supine position as previously described elsewhere [16, 17].

The research leading to this paper was driven by a concern for the quality of foetal outcomes in the presence of foetal head moulding. Foetal head moulding has been associated with severe damage to the newborn child's brain [1–3]. This form of CPD in which foetal head moulding is associated with severe brain damage may go unnoticed in low resource settings leaving the mother to suffer with a disabled child despite having had an apparently successful vaginal delivery [18]. CPD that is associated with severe brain damage may be even more devastating than the overt obstructed labour associated with death of the foetus [19], given the poor support structures for children with brain damage in low resource settings [18]. The objective of this study was to determine the associations between maternal anthropometric measurements maternal height and maternal pelvis height with recorded intrapartum foetal head moulding in Ugandan mothers with spontaneous vertex deliveries.

## 2. Materials and Methods

**Figure 2:**
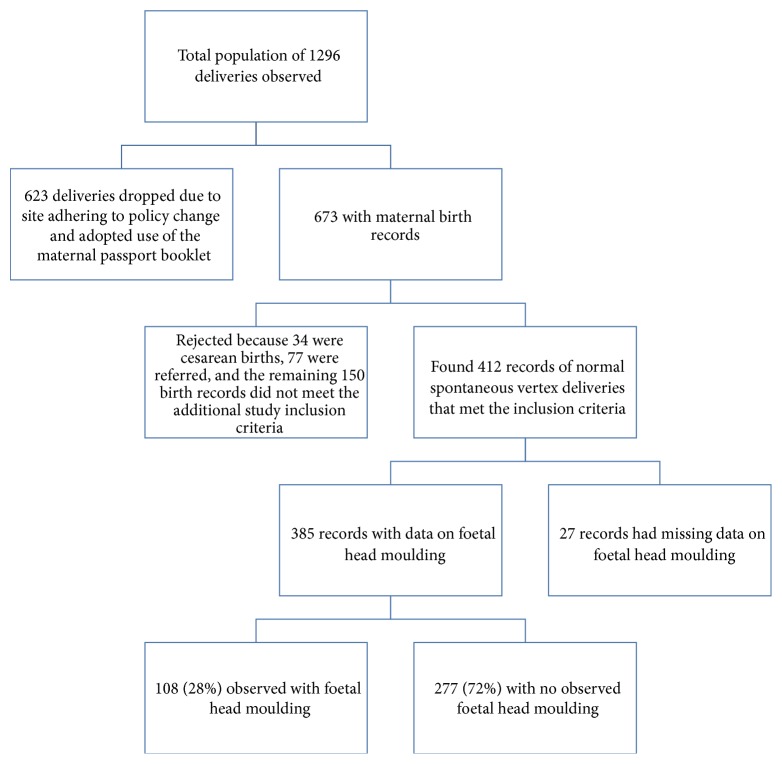
Flow diagram showing participant record selection.

This was a secondary analysis of data of 412 birth records from mothers whose active phase of labour was recorded on a WHO modified partogram. These 412 birth records meeting the study inclusion criteria were selected from a larger pool of 673 birth records obtained during the parent study (see Figure 2: flow diagram showing participant record selection). The selection criteria for mothers in the parent study included mothers identified as having an adequate pelvis by the attending midwife, carrying a singleton pregnancy, and in active phase of labour [17]. For this secondary analysis additional inclusion criteria were that the birth records had a minimum of two separate vaginal examinations and labour ending in spontaneous vaginal vertex delivery after the cervix reaching full dilatation. There were 217/412 (53%) records from Mulago hospital in central Uganda, 128/412 (31%) records from St. Joseph's hospital Kitgum in Northern Uganda, and 67/412 (16%) records from Kumi Hospital in Eastern Uganda. These sites were at the time of the parent study still retaining a copy of the partogram as part of the mother's birth records file.  Table 1 provides a summary of the descriptive statistics of the birth records on a selection of study variables.

The target sample size was 427 birth records of mothers who had normal deliveries. This was obtained using the sample size formula for cohort studies in the open EPI software (http://www.openepi.com) with the following assumptions: *α* = 95%, *β* = 0.05, and ratio of exposed to unexposed = 1. This gave a total of 328 records to obtain a minimum risk difference of 20% assuming that 50% of the unexposed had foetal head moulding. This value of 328 records was increased by a 1.3 allowance for the design effect at multiple sites to give a final target sample size of 427 birth records. For each record we obtained information on site, maternal age, maternal weight, gravidity, symphysis-fundal height, augmentation of labour, and the observation of foetal heart rate, cervical dilation, head descent, strength of uterine contractions, and status of the foetal membrane, whether raptured spontaneously or artificially, at each of the routine two hourly examinations. In addition data was extracted on the following: liquor staining, observed foetal head moulding, presence of caput succedaneum, birth weight, sex of baby, duration of labour, and APGAR score at 5 minutes, for each childbirth record. A record was also made of the maternal pelvis height by each of the participating midwives who had been trained in how to make this measurement using a pair of rigid rulers held at right angles with the point of one ruler on the most anterior and superior palpable point of the pubis bone of the pelvis and the other ruler on anterior superior iliac spines as shown in Figure 1 with mothers lying in the supine position as previously described [16, 17]. The main outcome variable, foetal head moulding, was recorded as presence or absence of foetal head moulding at any one of the serial vaginal examination recordings on the partogram.

Data was entered into Epidata version 3.2 (Epidata association, Denmark) and exported to STATA 12 (StataCorp LP, Texas, USA) for eventual analysis. Descriptive statistics were generated using means with additional comparisons for differences between sites using ANOVA. Multilevel logistic regression, using the gllamm function with Poison's distribution methods to control for clustering of repeated examinations on mothers at the different sites, was used to calculate the Incident Risk Ratios (IRRs) for foetal head moulding against the various predictor factors. Stepwise backward multilevel Poisson's regression was used to identify the significant variables of interest in the final model. Maternal height was retained in the final model as one of the key anthropometric measurements under study. During analysis records with missing values were dropped. The level of significance was set as *P* < 0.05 for all statistical tests.

Ethical considerations for the parent study [17] included obtaining ethical approval from the Makerere University School of Biomedical Science Institutional Review Board and the proposal for the larger study in which this one was nested was registered with the Uganda National Council of Science and Technology. As part of the approval process the study was registered with the office of the president, of the Republic of Uganda, and a letter of introduction was provided to inform the local leaders in the districts. The hospital administrators and heads of units were briefed of the parent study and the need to obtain a copy of the maternal birth records. All the participating nursing staff were verbally requested to be part of the study and offered an equivalent of 1 USD compensation for each birth record completed. Each mother in the parent study was informed about the study and requested to consent to participate in the study. With the exception of measuring maternal pelvis height there was no other procedure or modification made to the current birthing practice at any of the participating sites. No identifier marks of personal information were used in the analysis and reporting of the study results.

## 3. Results

In Table 1 foetal head moulding was observed in the recorded examinations of 28% of the birth records of mothers till childbirth. Of these records that observed foetal head moulding 75.64% remained with persistent foetal head moulding that once observed was seen at the subsequent recorded examinations for each mother till childbirth.

Table 2 compares the averages for of the following variables: age, height, weight, gravidity, fundal height, presence of caput succedaneum, presence of meconium staining, augmentation, birth weight, moulding, rupture of membranes, sex of baby, duration of active phase, and APGAR score at 5 mins and maternal pelvis height by each of the participating study sites. The table shows significant differences in the averages of all above variables with the exception of age of the mother and gender of the baby. In addition, 47% of birth records from Mulago hospital in Central Uganda reported foetal head moulding. This was significantly higher than what was observed at the other two sites: Kumi in Eastern Uganda 15% and Kitgum in Northern Uganda with 13% (*P* < 0.01).

Table 3 shows the results of the univariable Poisson's regression modelling analysis of the different study variables against the outcome of foetal head moulding. In this table the following were significantly associated with foetal head moulding: observed rupture of membranes, duration of active phase of labour, recorded cervical dilatation in centimetres, recorded foetal head descent in fifths, and the observed strength of uterine contractions at each examination till childbirth. In this Table 3, both the maternal height and maternal pelvis height were not significantly associated with foetal head moulding.

On multilevel multivariable Poisson's regression modelling the following previously significant variables become nonsignificant: cervical dilatation, foetal head descent, and strength of uterine contractions.  Table 4 shows the variables in the final model in which there was a 3% reduction in risk of foetal head moulding for each centimetre increase in maternal height. This, though not significant, was retained in the model as an anthropometric measurement (Adj. IRR 0.97 95% CI 0.97 to 1.00). There was a significant 22% reduction in risk of foetal head moulding for each centimetre increase in maternal pelvis height (Adj. IRR 0.88, 95% CI 0.80 to 0.97). There was also a significant 21% increase in the risk of foetal head moulding for each additional hour of labour (Adj. IRR 1.21, 95% CI 1.12 to 1.31), and a 90% increase in the risk of moulding was observed for each additional kilogram of birth weight (Adj. IRR 1.90, 95% CI 1.24 to 2.89). Presence of ruptured membranes had significant 10-fold reduction in risk foetal head moulding (Adj. IRR 0.10, 95% CI 0.03 to 0.32).

## 4. Discussion

We set out to determine the associations between maternal anthropometric measurements: maternal height and maternal pelvis height with intrapartum foetal head moulding in Ugandan mothers having spontaneous vertex deliveries. We found that only maternal pelvis height was associated with intrapartum foetal head moulding on final modelling. We found that for each centimetre increase in maternal pelvis height was associated with a significant 22% reduction in the risk of foetal head moulding compared with a borderline significant 3% reduction in the risk of foetal head moulding with each unit increase in maternal height, keeping all the other factors constant.

The significant reduction in risk of foetal head moulding associated with increasing maternal pelvis height (Table 4) could be explained by two observations: The first is based on the observations by Zivanovic (1968), who hypothesized that maternal pelvis height, then referred to as “*the level of the symphysis pubis,*” could influence the outcomes of labour due to its association with pelvis inclination [20]. According to this paper a smaller pelvis height would result in more direct vertical application of the descending foetal head on the maternal cervix. Proper application of the descending foetal head results in maximal transfer of net resultant force from the contracting uterine muscles on to the cervix leading to cervical effacement and dilatation [21]. Thus we would expect to see more rapid cervical dilatation and shorter duration of labour when the maternal pelvis height is least assuming that the maternal pelvis diameters are large enough for the descending foetal head.

This assumption of adequacy of the maternal pelvis diameters forms the basis of the second possible explanation for the significant reduction in the foetal head moulding with increasing maternal pelvis height. In Table 2 note records from Mulago hospital in central Uganda had the lowest mean maternal pelvis height and highest rate (47%) of foetal head moulding of the three study sites. This could be explained by variations in bone growth due to nutritional deficiencies. In populations with high levels of childhood malnutrition as it is observed in Sub-Saharan Africa, the growing girl child may fail to hit the maximal growth targets set by her genotype under optimal conditions [5, 9]. When this girl grows and eventually becomes pregnant under optimal feeding conditions she will carry a normal sized foetus. This sets the scenario for the series of events leading up to CPD as the mother attempts to push this big baby through her small pelvis [22, 23]. In a small study on dried rearticulated pelvis bones we observed that Maternal pelvis height is strongly correlated with the diameters of the mid and outlet segments of the birth canal [24]. This means that in mothers with the smaller values for maternal pelvis height from a population with high prevalence of childhood malnutrition we would expect shorter duration of labour and more foetal head moulding compared with mothers with larger values of maternal pelvis height (see Table 2). This suggests that maternal pelvis height could act as a possible measure of the maternal pelvis diameters for mothers in this setting with a high prevalence of childhood malnutrition. It is important to note that the presence of consultants at the abovementioned site (Mulago) might have resulted in higher rates of recorded foetal head moulding due to the subjective nature of this assessment. It is thus important to cautiously extrapolate the above observations to the general population.

Maternal height though retained in the final model was found to be nonsignificant (see Table 4). There are two possible explanations for this: the first is related to the small sample size of this study that may have been low for the risk ratio associated with height. Secondly, maximal height is dependent on the total growth achieved by the individual before closure of the growing ends of the long bones. This closure of the growing ends of the bones generally happens between the 16th and 20th years of life with the only exception being in the pubic bone of females where it occurs between the 28th and 30th years of life [25]. This means that maternal pelvis height, which is described above, may be a more sensitive measure of the diameters of the birth canal during the 2nd decade of a woman's life thus a better indicator of foetal head moulding compared with maternal height. This also supports the WHO's rejection of using maternal height as a screening tool for mothers prior to childbirth [14, 26]. Overall the 3% reduction in risk of moulding for each centimetre increase in height is still indicative of the importance of height especially as an indicator of chronic malnutrition [5]. Additional study with a larger sample size would be required to further identify clinically useful cut-off points for maternal height in relation to foetal head moulding.

Some of the limitations of this study include the small sample size for detecting the smaller risk ratios as was noted for maternal height and the additional challenges related to use of birth records in these settings [27]. Proper completion of these charts is especially challenging in busy and typically understaffed units. The last limitation is the challenge related to obtaining the anthropometric measurement whose accuracy may not be as high due to possible variability in the calibre and experience of people that run these units. To minimize these sources of bias the team ensured that all the nurses participating in the study were experienced at giving birth and received both the initial additional refresher training in how to measure the maternal pelvis height. In addition each record maternal pelvis height was recorded twice while foetal head moulding was limited to the very basic presence or absence of moulding due to the additional expertise required with the grading of moulding. The use of simulation techniques and multilevel modelling statistical methods to cater for the design effects helped narrow the confidence interval and variability in the number of records and measurements per mother and site. A final limitation was the change in policy to the use of maternal passport booklets that results in mother leaving the health facility with no complete record of the birth. The adoption of the maternal passport booklets resulted in leaving only a summary at the health facility and greatly reduced the potential sample population for the study. Whereas the sample size for this study was attained, future studies will need to adopt an appropriate strategy for the collection all the required information from the mothers in this setting.

In conclusion this study shows that increasing maternal pelvis height is associated with a significant reduction in intrapartum foetal head moulding. Increasing maternal height was associated with a nonsignificant reduction in risk of intrapartum foetal head moulding. This study shows that maternal pelvis height may be a good measure for the prediction of foetal head moulding by health workers in low resource settings thus aiding in early referral of mothers to the next level health facilities for better care. There is a need for more studies to standardise these measurements and identify clinically relevant cut-off values for use in various low resource settings as is seen in most of the remote and rural areas of Africa.

## Figures and Tables

**Table 1:** Descriptive statistics for the study population.

Variable (unit)	Total no. of records	Mean (SD)
Age (years)	401	25.66 (5.36)
Height (centimetres)	408	161.03 (6.78)
Weight (kilograms)	405	65.67 (9.37)
Gravidity	395	2.95 (2.13)
Symphysis-fundal height (centimetre)	400	37.76 (0.86)
Moulding (yes = 1, no = 0)	385	0.28 (0.37)
Sex of baby (male = 1, female = 0)	407	0.48 (0.50)
Birth weight (kilograms)	407	3.13 (0.47)
Apgar score at 5 mins	406	9.57 (0.72)
Pelvis height (centimetres)	402	6.97 (2.00)

**Table 2:** Showing the comparison of means for the different variables by study site.

Variable (units)	Mean (standard deviation)				ANOVA (*P* value)
Site	Overall	Mulago	Kitgum	Kumi	
Age (years)	25.65 (5.36)	25.48 (4.61)	25.72 (5.60)	25.89 (7.01)	1.71 (0.18)
Height (centimetres)	161.03 (6.76)	159.84 (7.09)	163.14 (5.60)	160.69 (6.88)	**107.78 (<0.01)**
Weight (kilograms)	65.67 (9.36)	66.99 (9.68)	66.64 (8.15)	59.94 (8.46)	**179.14 (<0.01)**
Gravidity	2.95 (2.11)	2.39 (1.21)	3.30 (2.36)	3.94 (3.09)	**182.72 (<0.01)**
Fundal height (centimetres)	37.76 (0.86)	37.95 (0.55)	37.57 (1.04)	37.55 (1.12)	**118.12 (<0.01)**
Presence of caput succedaneum (yes = 1, no = 0)	0.07 (0.27)	0.06 (0.24)	0.05 (0.24)	0.15 (0.38)	**42.71 (0.01)**
Meconium staining (yes = 1, no = 0)	0.11 (0.37)	0.11 (0.33)	0.06 (0.27)	0.26 (0.58)	**71.66 (<0.01)**
Augmentation (yes = 1, no = 0)	0.05 (0.21)	0.04 (0.18)	0.06 (0.22)	0.07 (0.26)	**7.71 (<0.01)**
Birth weight (kilograms)	3.14 (0.47)	3.22 (0.47)	3.12 (0.37)	2.96 (0.57)	**94.37 (<0.01)**
Moulding (yes = 1, no = 0)	0.28 (0.40)	0.47 (0.41)	0.13 (0.25)	0.15 (0.30)	**189.04 (<0.01)**
Rupture of membranes (yes = 1, no = 0)	0.30 (0.27)	0.27 (0.50)	0.36 (0.23)	0.26 (0.19)	**11.19 (<0.010)**
Sex of baby (male = 1, female = 0)	0.48 (0.50)	0.48 (0.50)	0.48 (0.50)	0.46 (0.50)	0.54 (0.58)
Duration of active phase (hours)	6.85 (2.30)	6.36 (1.79)	7.81 (3.21)	6.52 (1.66)	**34.58 (<0.01)**
Apgar score at 5 mins	9.57 (0.72)	9.56 (0.67)	9.64 (0.78)	9.42 (0.71)	**20.23 (<0.01)**
Pelvis height (centimetres)	6.97 (2.00)	5.75 (1.46)	7.71 (1.42)	9.35 (1.18)	**204.89 (<0.01)**

**Table 3:** Showing univariate regression modelling of foetal head moulding by the study variables.

Variable	Univariate modelling: IRR (95% CI, *P* value)
Age	1.02 (0.98 to 1.05, 0.39)
Height (cm)	1.00 (0.98 to 1.03, 0.96)
Weight (kgs)	1.01 (0.99 to 1.03, 0.22)
Gravidity	1.01 (0.92 to 1.10, 0.89)
Fundal height	1.05 (0.84 to 1.32, 0.63)
Presence of caput succedaneum (yes = 1)	1.42 (0.77 to 2.66, 0.26)
Meconium staining	0.89 (0.52 to 1.55, 0.70)
Induction	1.18 (0.52 to 2.68, 0.70)
Birth weight (kgs)	1.38 (0.93 to 2.06, 0.11)
Foetal heart rate	1.01 (0.98 to 1.04, 0.40)
Ruptured membranes	0.12 **(0.04 to 0.34, <0.01)**
Sex of baby (male = 1)	1.28 (0.89 to 1.84, 0.19)
Duration of active phase of labour	1.17 **(1.11 to 1.23, <0.01)**
Cervical dilatation	1.93 **(1.47 to 2.55, <0.01)**
Foetal head descent	0.53 **(0.41 to 0.69, <0.01)**
Strength of uterine contractions	2.59 **(1.65 to 4.04, <0.01)**
Apgar score at 5 mins	0.97 (0.75 to 1.23, 0.79)
Pelvis height (cm)	0.92 (0.84 to 1.01, 0.08)

**Table 4:** Multivariate regression model for foetal head moulding by the different study variables.

Variable	Univariate model IRR	Final multivariate model: IRR (95% CI, *P* value)
Height (cm)	1.00	0.97 (0.97 to 1.00, 0.05)
Birth weight	1.38	1.90 **(1.24 to 2.89, <0.01)**
Ruptured membranes	0.12	0.10 **(0.03 to 0.32, <0.01)**
Duration of active phase	1.17	1.21 **(1.12 to 1.31, <0.01)**
Pelvis height (cm)	0.92	0.88 **(0.80 to 0.97, 0.01)**

## Authors' Information

Ian G. Munabi is Trained Clinical Anatomist with wide ranging experience and practice in various clinical settings. Samuel Abilemech Luboga is a Trained Surgeon and Professor of Anatomy with several years of experience of working in various clinical low resource settings. Livingstone Luboobi is a Professor of Mathematics with several years of experience in applying various mathematical concepts to help understand various health related challenges. Florence Mirembe is a Professor of Gynaecology and Obstetrics with several years of working experience and clinical practice in low resource settings.
